# EGF increases expression and activity of PAs in preimplantation rat embryos and their implantation rate

**DOI:** 10.1186/1477-7827-5-4

**Published:** 2007-01-29

**Authors:** Eliahu D Aflalo, Uriel A Sod-Moriah, Gad Potashnik, Iris Har-Vardi

**Affiliations:** 1 Department of Life Sciences, Ben-Gurion University of the Negev, P.O.Box 653, Beer-Sheva 84105, Israel; 2 Fertility and In vitro Fertilization (IVF) Unit, Department of Obstetrics and Gynecology, Soroka University Medical Center, Faculty of Health Sciences, Ben-Gurion University of the Negev, Israel

## Abstract

**Background:**

Embryo implantation plays a major role in embryogenesis and the outcome of pregnancy. Plasminogen activators (PAs) have been implicated in mammalian fertilization, early stages of development and embryo implantation. As in-vitro developing embryos resulted in lower implantation rate than those developed in-vivo we assume that a reduced PAs activity may be involved.

In the present work we studied the effect of EGF on PAs activity, quantity and embryo implantation.

**Methods:**

Zygotes were flushed from rat oviducts on day one of pregnancy and grown in-vitro in R1ECM supplemented with EGF (10 ng/ml) and were grown up to the blastocyst stage. The control groups were grown in the same medium without EGF. The distribution and quantity of the PAs were examined using fluorescence immunohistochemistry followed by measurement of PAs activity using the chromogenic assay. Implantation rate was studied using the embryo donation model.

**Results:**

PAs distribution in the embryos was the same in EGF treated and untreated embryos. Both PAs were localized in the blastocysts' trophectoderm, supporting the assumption that PAs play a role in the implantation process in rats.

EGF increased the quantity of uPA at all stages studied but the 8-cell stage as compared with controls. The tissue type PA (tPA) content was unaffected except the 8-cell stage, which was increased. The activity of uPA increased gradually towards the blastocyst stage and more so due to the presence of EGF. The activity of tPA did not vary with the advancing developmental stages although it was also increased by EGF.

The presence of EGF during the preimplantation development doubled the rate of implantation of the treated group as compared with controls.

## Background

The major obstacle in IVF treatments is the low embryo implantation rate. The composition of the embryo culture medium supposed to mimic the physiological environment, addition of growth factors to the medium may be vital not only for improving the embryo development but also for increasing the embryo implantation rates in IVF programs. Plasminogen activators are members of one of the main enzyme family that participate in embryo implantation.

Plasminogen activators (PAs) and matrix metalloproteinases (MMPs) have been implicated in mammalian gametogenesis [1], ovulation [2,3], fertilization [4,5], early development and embryo implantation [3,6,7]. The PAs are serine proteases, which convert the inactive plasminogen to the potent protease plasmin. Plasmin can degrade directly or indirectly, through the activation of metalloproteinase zymogens, all components of the extracellular matrix [8,9].

There are two types of PAs, the tissue-type plasminogen activator (tPA) and the urokinase-type plasminogen activator (uPA). Plasminogen, its activators and inhibitors participate in the implantation process. Trophoblast cells of mouse blastocysts cultured in-vitro produced PAs during the period corresponding to the in-vivo invasion into the endometrium [10]. In embryos of the homozygous tw73 mouse mutant, PAs were reduced and were concomitantly associated with implantation failure [11]. The invasion of trophoblast cells during the implantation process could be blocked by inhibitors of serine proteases, illustrating the role of these enzymes in the invasion process [12,13].

In the human, embryo implantation is considered to be one of the most critical steps in reproduction. An estimated 30–70% of embryos are lost prior to or during implantation [14]. In in-vitro fertilization-embryo transfer (IVF-ET) programs only 12% of the transferred embryos successfully implant [15].

In the implantation process, two major factors participate: the uterus undergoes changes that prepare it for the arrival and implantation of embryos, and the embryos which undergo cellular reorganization that enables them to penetrate the endometrium and to form the placenta. We assume that one of the reasons for low implantation rate of in-vitro developing embryos involves reduced PAs activity.

Preimplantation embryos express variety of growth factors and their receptors [16]. These include Epidermal Growth Factor (EGF) receptors, which are expressed during the preimplantation stage by murine [17], porcine [18] and human embryos [19]. Many attempts have been made to determine whether preimplantation development, cleavage, and differentiation of the blastocyst are influenced by either endogenous or exogenous growth factors.

Among these growth factors, EGF has been shown to stimulate both cellular proliferation and differentiation [20]. Several reports demonstrated the effect of EGF as a mitosis-promoting agent on perimplantation embryo development. EGF was found to improve the preimplantation embryo development by increasing cell metabolism and proliferation [21]. In media enriched with EGF (and other factors of the EGF family), higher blastocyst rates as well as improved trophectoderm expansion were demonstrated [16]. It was found that addition of EGF to mice cultured preimplantation embryos stimulated DNA and RNA synthesis and increased cell numbers [21]. Translational inhibition of the EGF-receptor mRNA by anti-sense RNA treatment leads to a delay in blastocyst formation [22]. However, very few studies on the effect of EGF during preimplantation development of embryos of species other than mouse are reported.

Despite the beneficial effects of growth factors on preimplantation embryo development, more recent medium formulations, particularly those containing amino acids, but no growth factors, have resulted in rates of development that approximate those occurring in-vivo, with nearly 100% of embryos forming blastocysts [23,24]. These embryos have an increased cell number, develop a primitive endoderm, and the levels of gene expression are quantitatively and qualitatively similar to those in in-vivo grown embryos [25].

In this study we examined the effects of exogenous EGF on the expression and activity of tPA and uPA in preimplantation embryos developed in-vitro, and we compared the implantation rate of these embryos to in-vitro developed once that were unexposed to EGF.

## Methods

The following study was approved by the Institutional committee for animal care and ethics at Ben-Gurion University of the Negev, Beer-Sheva, Israel, before the commencement of the experiments.

### Animals

Mature female Wistar rats 2–3 months old, weighing 180–230 g were used. The animals were kept in a temperature-controlled room maintained at 22–24°C with lighting regimen of 14 hours light 10 hours dark (light on 5:00 AM – 7:00 PM). The rats were allowed free access to rat chow and tap water.

Daily vaginal smears were taken at 10:00 AM, and the stage of estrous cycle was determined. Overnight caging of a proestrous female with a male of proven fertility induced pregnancy. The next day, the presence of a vaginal plug or spermatozoa in the vaginal smear was designated as day 1 of pregnancy.

Pseudopregnancy was induced by electrical stimulation of the cervix at proestrous, as reported earlier [26]. The next day was considered day 1 of pseudopregnancy.

### Collection of embryos

Zygotes were flushed with rat 1-cell embryo culture medium (R1ECM) [26] from oviducts at day-1 of pregnancy. All equipment and media used were sterile. Ovary-oviduct complexes were removed from anesthetized animals. The complexes were placed in R1ECM, and the oviducts were separated under a dissecting microscope. 30-gauge blunt end needle attached to a syringe containing R1ECM was inserted through the oviductal end held by forceps surrounding needle and tube. Zygotes in their cumulus mass were flushed and the cumulus cells were removed by gentle aspiration through a micropipette 150–200 μm in diameter several times in R1ECM containing 80 U/ml of hyaluronidase. Clean zygotes were washed 3 times by transferring them into fresh R1ECM to remove traces of hyaluronidase. Flushed zygotes were collected with a mouth-controlled micropipette 150–200 μm in diameter and transferred to the culture dishes.

### Embryo culture

Clean zygotes were grown in-vitro to the developmental stages of two-cell, four-cell, eight-cell, morula and blastocyst. Each group of embryos consisted of 25–35 embryos collected from six pregnant females. This was repeated three times for each developmental stage (total of about 90 embryos per stage). Groups of 25–35 zygotes were placed into 35-mm-diameter culture dishes (Nunc Co., Denmark) containing 50 μl of R1ECM medium under a layer of mineral oil (previously equilibrated to the experimental conditions) and cultured at 37°C under 5% CO2 in air (control group). This medium was shown by Miyoshi et al. [26] to enable cultured rat embryos to reach the blastocyst stage. In comparing various media, at our laboratory R1ECM was found to be the best medium to enable a synchronous development of embryos (95% of total) to the blastocyst stage. The developing embryos seemed to be normal in their morphology, with almost no fragmentation.

In the experimental group, EGF (Sigma) was added to the medium in a final concentration of 10 ng/ml. In every developmental stage embryos from control and experimental group were collected, washed 3 times in fresh R1ECM and used for immunofluorescence study. Three pools of 50–60 embryos at the developmental stages of 2-cell, 8-cell, morula and blastocyst were collected in 50 μl aliquots of fresh R1ECM and stored at -70°C for PAs activity assay.

### Embryo immunohistochemistry

The method used was basically that of Dubey et al. [27] with various modifications. Groups of 20–25 embryos at different developmental stages from the experimental and control groups were fixed in 4% paraformaldehyde in phosphate buffered saline (PBS) at room temperature and washed twice in PBS, PH 7.4 for 5 minutes. Five percent bovine serum albumin (BSA) in PBS was used for dilution of antibodies and washings (PBS-BSA). The embryos were washed four times in PBS-BSA before immunoreactions. Embryos, randomly chosen were either exposed to polyclonal rabbit anti rodent uPA or rabbit anti rat tPA (American Diagnostics, Pendelton, IN) at a concentration of 4 μg/ml. Embryos were then incubated overnight in 50 μl of each antibody solution under paraffin oil in a 35 mm culture plate in a moist chamber at 4°C. The embryos were then washed four times in PBS-BSA and incubated with Cy3-conjugated goat anti-rabbit IgG (Jackson ImmunoResearch Laboratories, Inc., West Grove, PA) at 37°C for 60 minutes. The conjugated antibody was used at a dilution of 1:300 in PBS-BSA. After incubation with the secondary antibody, the embryos were washed again in PBS-BSA and stained with DNA stain 4', 6-diamidino-2-phenylindole (DAPI) (Vector Laboratories, Burlingame, CA) and mounted in Flouromount – G (Southern Biotechnology Associates, Inc. Birmingham, AL) to minimize quenching. To confirm that the fluorescence observed was neither attributable neither to nonspecific binding of the secondary antibody nor to formaldehyde-induced autofluorescence, negative controls (without primary antibody) were established during each immunoreaction procedure. The immunocytochemistry staining procedure was repeated three times for each stage of embryo development on different batches of embryos.

### Image analysis

The distribution and concentration of PAs in the embryos were visualized by fluorescent microscopy on a Zeiss laser scanning confocal microscope equipped with an ×100 objective. Z-sections and XZ-sections were obtained from 3D scanning by using LSM510 software (Zeiss, Feldbach, Switzerland). The PAs density for each embryo was computed by image analysis based on the same principles as manual counting described elsewhere [27]. The embryos' fluorescent images were downloaded using image analysis software, ImagJ (NIH, Bethesda, MD). These images were stored in the computer by use of pixels. All the slices obtained from 3D scanning were of 0.7 μm and were analyzed by counting the number of pixels of the Cy3 (red color) in the whole slice. Total pixels in a whole embryo were calculated by summing the number of pixels in all the slices of an embryo. This method showed the total amount of PAs expression in each embryo. The number of pixels in an embryo represents the intensity of PAs staining (fluorescence), which is in turn proportional to the amount of PAs in that embryo. Each experimental group consisted of 8–10 embryos and the measurements repeated three times with different batches of embryos from each developmental stage stained at different times (total number of 24–30 embryos per stage).

### Enzymatic assay for plasminogen activators

A chromogenic assay, as described originally by Coleman and Green [28] with some modifications to enable measurement of embryonic PAs' activities was used. This assay measures total plasminogen activator activity without differentiating between tPA and uPA. The uPA inhibitor amiloride was used to differentiate between the relative activity of uPA and tPA.

Three frozen 50 μl aliquots, from each stage, containing a total of approximately 160 embryos were thawed, pooled, and lysed by repeated freezing and thawing in R1ECM. Each such pool was divided to 4 aliquots (containing lysed material equivalent to 37–40 embryos). Lysed embryos and endometrial extract samples were mixed with, or without (negative control) 2.5 μg plasminogen and with or without the uPA inhibitor amiloride (Sigma, St. Louis, MO) to reach a final volume of 50 μl. The mixture was incubated at 37°C for 60 minutes. At the end of this incubation, 1 ml of a solution, containing 220 μM 5,5'-dithiobis (2-nitrobenzoic acid) and 220 μM thiobenzyl benzylcarboxycarbonyl-L-lyzinate (Sigma) in phosphate buffer was added, and the incubation was continued for another 60 minutes. The absorbance at 410 nm was indicative of PAs activity. Standard curves of uPA activity (American diagnostics), ranging from 0.02 to 4 mIU, were included in each assay to estimate PAs activity. The sensitivity of the assay under the conditions described above was 0.02 ± 0.01 mIU. No PAs activity was detected in the absence of plasminogen (negative control). The above measurements were repeated three times for each experimental group.

### Embryo transfer

Embryo transfer was done as previously described by Hogan et al. [29] and by Ertzeid and Storeng [30]. Seventeen pseudopregnant female rats at day-5 of pseudopregnancy were anesthetized by i.p. injection of chloral hydrate 10% (w/v; 0.003 ml/g body mass) and the uterine horns were exposed via lateral incisions. Blastocysts to be transferred were collected from EGF supplemented in-vitro grown blastocysts or controls. Blastocysts were collected with a mouth-controlled pipette with a curved tip, 150–200 μm in diameter. The tip of the pipette was inserted into a hole made previously by inserting 23-gauge needle through the uterine wall at the oviductal end. Five EGF treated or 5 controls were transferred in a minimal volume of 5 μl, at random, into either of the contralateral uterine horns of the pseudadin-77@smile.netopregnant recipient. Thereafter, the uterine horns were replaced in the abdominal cavity. After suturing the abdominal muscles, the skin incision was closed using metal clips. The recipients were euthanized by i.p. overdose injection of chloral hydrate. Implantation rate was determined by counting implanted embryos 9 days after transfer.

### Statistical analysis

Data are expressed as means ± SEM. Statistical analysis was performed using paired "t"-test or two-way analysis of variance (ANOVA) followed by the least significant differences (LSD) test for multiple comparisons. P < 0.05 was defined as statistically significant difference.

## Results

### Immunolocalization of tPA and uPA in preimplantation embryos; effect of EGF

**Figure 1:**
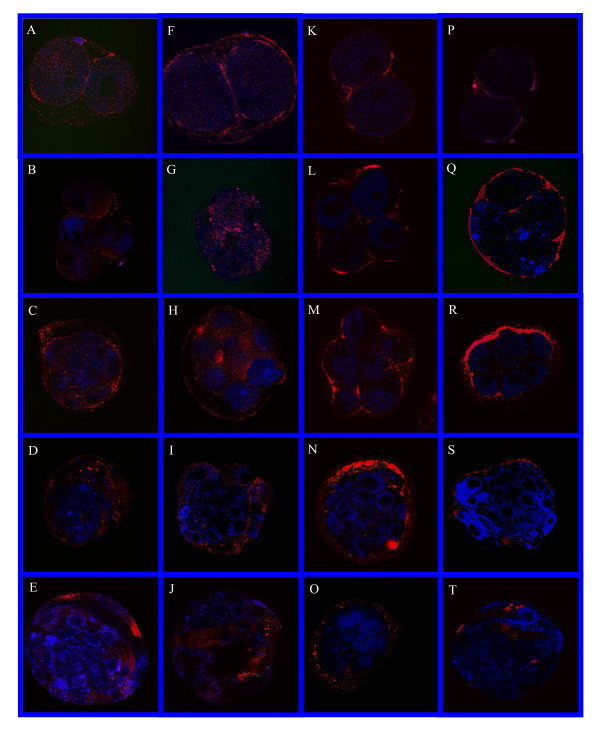
Immunolocalization of uPA and tPA in preimplantation developing rat embryos grown in-vitro with and without the presence of 10 ng/ml EGF in the medium. (A, F, K, P), 2-cells. (B, G, L, Q), 4-cells. (C, H, M, R), 8-cells. (D, I, N, S), Morula. (E, J, O, T), Blastocyst. (A-E), uPA -EGF. (F-J), uPA +EGF. (K-O), tPA -EGF. (P-T), tPA +EGF.

Immunohistochemical staining for the location of tPA and uPA in preimplantation embryos developed in-vitro with or without exogenous EGF are shown in figure 1. Plasminogen activators were detected in all the embryonic developmental stages in-vitro (from the two cell stage to the blastocyst stage) both in the presence of exogenous EGF and in controls (Fig. 1A-T). The uPA was detected in the cell cytoplasm and plasma membrane (Fig. 1A-J) while tPA was detected on the cell membrane and in the perivitelline space (Fig. 1K-T). In the blastocyst stage, both with and without EGF, PAs were localized mainly in the trophectoderm (Fig. 1E,J,O,T). There were no differences in PAs localization in the presece of EGF, between any of the stages of development.

### Effect of exogenous EGF on the level of PAs expression at different embryonic developmental stages

**Figure 2:**
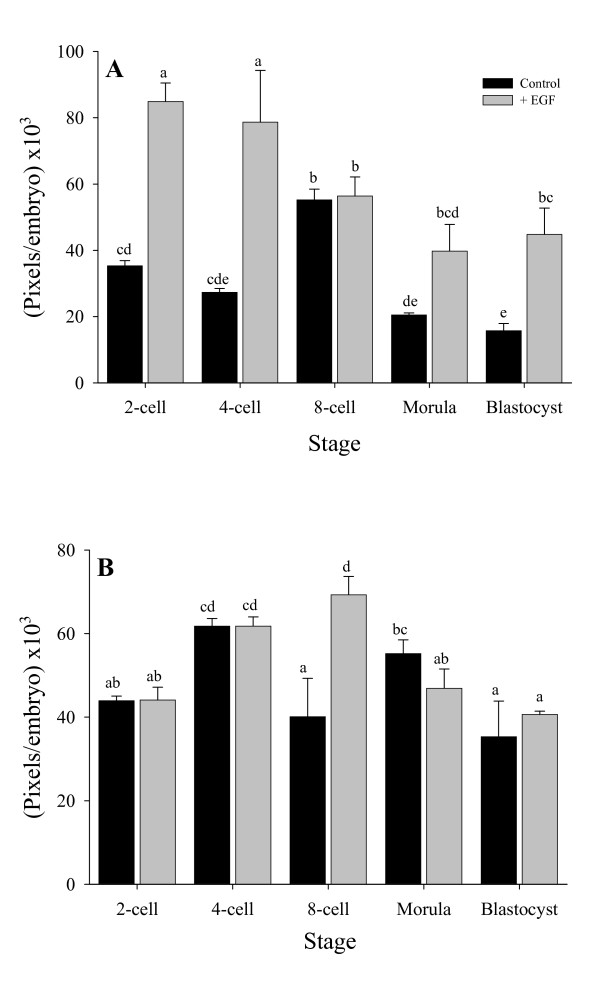
Quantitative (Pixels/embryos) uPA expression in preimplantation rat embryos developed in-vitro with and without the presence of EGF in the medium (A). Quantitative (Pixels/embryos) tPA expression in preimplantation rat embryos developed in-vitro with and without the presence of EGF in the medium (B). Means ± SEM. Different letters above the columns represent significant differences (ANOVA followed by LSD test, P < 0.01).

Quantitative measurment of total uPA in a whole embryo at different developmental stages showed significantly higher expression in in-vitro developed embryos in the presence of exogenous EGF in the 2-cell, 4-cell, morula and blastocyst stages compared to the control groups (140%, 188%, 94% and 185%, respectively. P < 0.01) (Fig. 2A). In contrast, the highest expression of uPA in the control developed embryos was found at the 8-cell stage (55.2 × 103 Pixels per embryo). In the presence of exogenous EGF, highest expression of uPA was found at the 2-cell stage (84.9 × 103 Pixels per embryo) which then decreased gradually towards the blastocyst stage (44.75 × 103 Pixels per embryo) (Fig. 2A).

Total tPA expression in a whole embryo showed highest expression in embryos developed in the presence of exogenous EGF at the 8-cell stage in comparison to the controls (69.28 and 40.1 × 103 Pixels per embryo, respectively, see Fig. 2B). In all the other developmental stages the addition of exogenous EGF did not affect the total amount of tPA in the cultured embryos.

### The effect of exogenous EGF on the activity of PAs in preimplantation embryos

**Figure 3:**
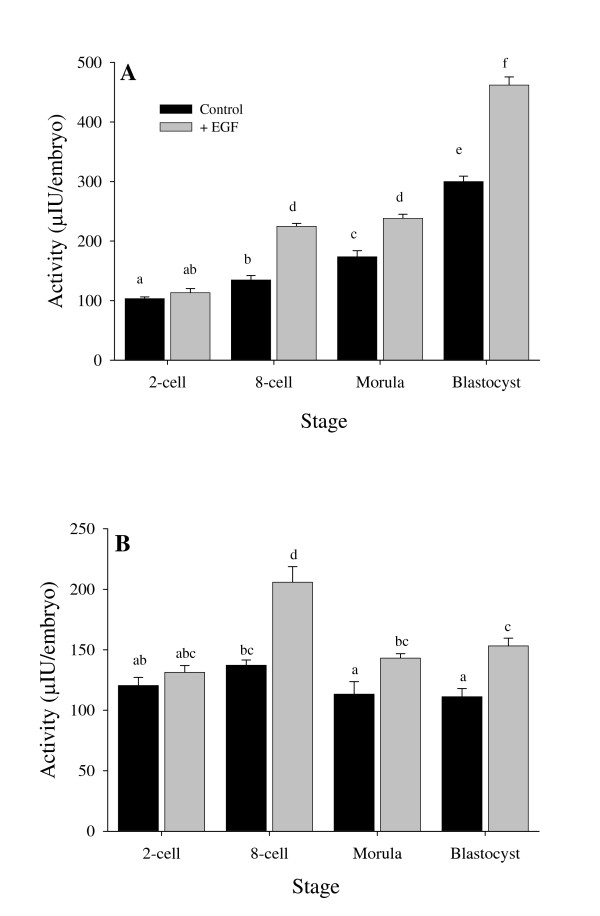
Activity of uPA in preimplantation embryos developed in-vitro with and without the presence of EGF in the medium (A). Activity of tPA in preimplantation embryos developed in-vitro with and without the presence of EGF in the medium (B). Means ± SEM. Different letters above the columns represent significant differences (ANOVA followed by LSD test, P < 0.01).

The uPA activity (fig. 3A) in the control embryos was low in the 2-cell stage (103.28 μIU/embryo) and increased gradually towards the blastocyst stage (299.86 μIU/embryo, p < 0.01). The same pattern of increase in uPA activity was found in the presence of exogenous EGF with significantly higher activity in 8-cell, morula and blastocyst stages (66.7%, 37.3% and 54% respectively, p < 0.05) (Fig. 3A).

The tPA activity did not change significantly during in-vitro embryo development of the control (111.24–137.16 μIU/embryo, p < 0.05, see Fig. 3B).

At the 8-cell stage there was a significant increase in tPA activity in embryos developed with the addition of exogenous EGF (205.84 μIU/embryo, p < 0.05, see Fig. 3B).

### Effect of exogenous EGF on embryo implantation rate

**Figure 4:**
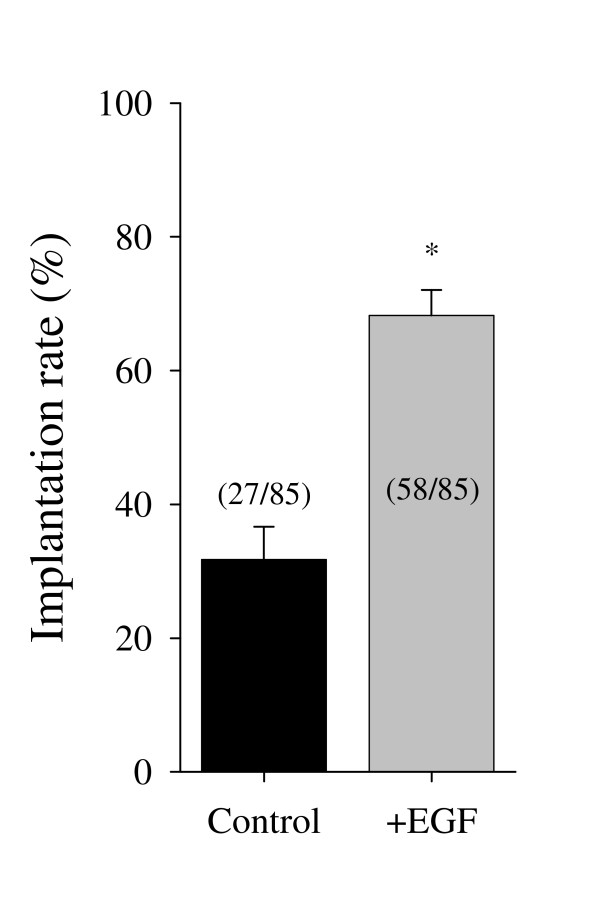
Percentage of implanted embryos in contralateral uterine horns of 17 pseudopregnant rats into which in-vitro developed blastocysts with and without the presence of EGF in the medium were transferred. Means ± SEM. In parentheses, number of implanted embryos out of transferred blastocysts. * = P < 0.01 (Paired "t"-test).

The implantation rate of in-vitro developing blastocysts, with or without the presence of exogenous EGF, was studied using 17 pseudopregnant rats. Five developed blastocysts from each experimental group were transferred into each of the contralateral uterine horns (total of 85 embryos from each group). The implantation rate of the control developing blastocysts was significantly low as compared with that of blastocysts developed in the presence of exogenous EGF (31.7% and 68.2% respectively, p < 0.01. Fig. 4)

## Discussion

The purpose of this study was to examine the effect of EGF on PA proteins expression (cellular distribution and quantity) and activity in in-vitro developing rat preimplantation embryos.

In our previous study we found that in rat embryos, uPA and tPA were present in all the stages of preimplantation development [24]. Other previous animal studies have demonstrated differential expression of PAs activity in cultured mouse embryos with greatest expression in early gestation [31]. Also, Zhang *et al*. [32] reported expression of uPA gene and uPA activity in preimplantation rat embryos developed in-vitro. Khamsi *et al*. [14] reported the presence of mRNA for uPA in human blastocysts. These cumulated observations hypothesize that PAs may be involved in extracellular matrix modification during preimplantation embryo development and implantation. However, information about the expression and immunolocalization of uPA and tPA have not been reported in in-vitro preimplantation developing rat embryos grown in EGF supplemented culture medium.

Quantitative measurements of PAs expression in a whole embryo enabled us to compare embryos at different developmental stages and different growth conditions as shown in our previous study [24]. Immunoreactive uPA was present in the embryonic cell cytoplasm and plasma membranes in all developmental stages, grown with or without EGF in the medium, while tPA was detected on the plasma membranes and in the perivitelline spaces. These results are supported by Zhang *et al*. [32] who showed that PAs activity was detected in oocytes and embryos between the 2-cell and blastocyst stages. In blastocysts developed in-vitro, with or without EGF, PAs were localized mainly in the trophectoderm and these findings are in agreement with previous observations, showing an increase in uPA activity towards the blastocyst stage in in-vivo and in in-vitro developing embryos [33]. The presence of uPA in the trophectoderm combined with its high activity in this stage supports the idea that blastocyst uPA activity is important for implantation, thus, inhibition of PAs decreases the extent of trophoblast attachment and outgrowth in-vitro [34]. Sappino *et al*. [7] showed that trophoblast cells grown in-vitro possesed PAs activity at the normal time of their penetration into the endometrium in-vivo. The uPA was the major enzyme secreted from trophectoderm cells with the highest activity on days five to seven of pregnancy. In addition, in implantation-defective rats, uPA activity and extra-cellular matrix degradation were reduced as compared with intact animals [11]. All the above support the assumption that uPA activity is important for the implantation process.

Pro-uPA is synthesized as an inactive single chain that can be stored or secreted. The secreated pro-uPA can be cleaved to produce the two-chain active molecule uPA, by the aid of limited proteolytic activity of plasmin [35]. Pro-uPA or uPA can be found free in the cytoplasm and the extracellular matrix or bound to a membrane uPA receptor [36]. Whether the uPA identified in this study is pro-uPA or uPA is unknown since the immunofluorescence staining method used cannot distinguish between the two. There is no correlation between the level of activity and the measured quantity of uPA in either EGF treated embryos or in the control ones. This could be explained by yet undetermined variations in the ratio of active and inactive forms of uPA.

The in-vivo conditions within the oviduct and uterine cavity constitute the optimal environment providing not only proper temperature, pH and ion concentrations, but also the crucial amount of nutrients and growth factors [37]. Studying animal models suggested that growth factors are embryotrophic [38] and EGF may promote preimplantation embryo growth [39,40], as well as trophoblast invasion and postimplantation embryo growth [41,42]. In addition, growth factors' receptors are localized on blastomeres of mammals preimplantation embryos [17,43,44]. These receptors appear in the apical blastomeres' membrane of the 4-cell stage mouse embryos [17]. Several reports have demonstrated the effect of EGF on embryo development, but very little is known about its effect on the ability of in-vitro developing embryos to implant.

The activity of PAs system in various tissues is under hormonal and cytokine control. Because angiogenesis requires proteolysis, several angiogenetic factors have been shown to promote activity of the PAs system. Kasza and Koj found that EGF enhances uPA, tPA and PAI-1 expression in astrocytoma cells [45]. In addition, Li *et al*. showed that in endothelial cells and monocytes uPA up-regulates itself via its EGF domain and uPA receptor [46]. The expression of EGF and its receptor in trophoblasts together with the marked proteolytic activity in the placental bed suggests an interaction between the two systems; therefore we studied the effect of EGF on the activity of PA enzymes in preimplantation embryos, and its effect on implantation rate.

In the present study, preimplantation embryos grown in-vitro with EGF, showed a quantitative increase in PAs expression. There was a significant increase in the quantity of uPA in all developmental stages, except the 8-cell stage. The addition of EGF did not change the cellular distribution of PAs in the in-vitro developing preimplantation embryos. At the 8-cell stage only, there was an increase in tPA quantity. The data hypothesize that EGF affects embryonic PAs expression and activity in a stage specific and activator (uPA or tPA) specific manner. The significant increase in embryonic uPA content detected in the 2-cell stage, in the presence of EGF, hypothesize the recognition of EGF at this early developmental stage by apical blastomeres membrane receptor in contrast to the finding of Weliy *et al*. [17] who reported presence of EGF receptors from the 4-cell stage.

The importance of uPA for the process of implantation is re-enforced by its relocalization in the trophectoderm cells. Despite the increased concentration of tPA in the trophectoderm cells, its activity in the blastocyst stage is reduced, raising the hypothesis that it may be in an inactive enzyme form in this stage which is indistinguishable in the immunostaining method used [24].

Embryonic PAs activities were detected in all the stages of the in-vitro preimplantation embryo development in either EGF treated or untreated embryos. The embryonic genome of rats and mice starts to be expressed at the 2-cell stage [47] and the presence of PAs already in this early stage may be a consequence of this activation. The enzyme tPA attaches to a cell membrane receptor, annexin II, thus directing the proteolytic activity to selected extracellular regions [48]. Such attachment of tPA from maternal source could also, at least partially, contributes to the presence of tPA found in the early developmental stages.

The embryonic extracellular matrix is in a continuous turnover during the embryonic development. The 8-cell stage is characterized by structural changes taking place in the embryo during the compaction process. It is therefore likely that the tPA effect on the remodeling taking place in the 8-cell stage is more sensitive to the presence of EGF. The increase in tPA expression from the 2-cell stage to the 4-cell stage in both EGF treated and untreated embryos, is probably due to de novo synthesis of tPA since there is no extraembryonic tPA source but the embryos in the culture. The increased tPA activity at the 8-cell stage could be due to the increased immunoreactive tPA quantity measured at this stage.

The uPA activity gradually rises from the early developmental stages up to the blastocyst stage. Such high activity in the blastocyst stage at the normal time of nidation and implantation may indicate its importance in these processes. These data suport the finding of Axelrod regarding a correlation between aberrant implantation in rats and low uPA activity and extra-cellular matrix degradation as compared with intact animals [11]. In addition, Kubo *et al*. [34] showed that inhibition of PAs activity prevents the adhesion of mice embryos to decidual cells grown in-vitro.

Higher PAs activity was observed in in-vitro developed embryos in the presence of EGF as compared with control ones. This could be explained by a reduced metabolic activity in the control developed embryos [49].

The present results confirm the importance of EGF in modulating the PA/plasmin system in the embryo during preimplantation development. Wiley *et al*. [17] showed expression of EGF receptor mRNA in preimplantation embryos which hypothesize that EGF may act directly on embryos, perhaps in a paracrine manner. These observations suggest specific regulation of proteinase activity in blastocyst that is independent on the mitogenic effects of EGF. Upon visual inspection, the sizes of blastocysts were similar in EGF-treated and untreated cultures. This does not entirely exclude the possibility that increased expression of proteinase activity could be due increased cell numbers (proliferation) in response to EGF.

Previous work demonstrated significant lower implantation rate of in-vitro developed embryos compared to in-vivo ones in the rat [33]. In this work we have compared the implantation rate of in-vitro developed blastocysts with or without the addition of EGF. Culturing the embryos in-vitro with EGF significantly increased their implantation rate. This finding is in accordance with Morita *et al*. [50] who showed a significant increase in implantation rate of in-vitro developed mice blastocysts treated with EGF.

Our data suggest that development in-vitro, which is morphologicaly indistinguishable from that in-vivo, may impair embryo quality and thus reduce implantation rate, independent of the endometrial condition of the recipient. This embryo donation model may provide a way of determining whether a developmental failure may be a result of the in-vitro conditions. It is conceivable that the presence of EGF in the reproductive tract would enhance the quality of embryos developed in-vivo. Indeed, oviduct epithelium is known to promote embryogenesis in many mammalian species and the expression of EGF in the oviduct and its involvement in early embryo development has been reported [51-53].

## Conclusion

In conclusion, addition of EGF to the culture medium increases uPA activity and embryo implantation rates in the rat.

## Authors' contributions

EDA participated in the planning of the project, carried out the animal experimentation, immunohistochemistry, image analysis studies and participated in preparation of the manuscript. USM participated in the planning of the project, animal experimentation and participated in preparation of the manuscript. GP participated in preparation of the manuscript. IHV participated in the planning of the project, statistical analysis and in preparation of the manuscript.
